# A Manual Curation Strategy to Improve Genome Annotation: Application to a Set of Haloarchael Genomes

**DOI:** 10.3390/life5021427

**Published:** 2015-06-02

**Authors:** Friedhelm Pfeiffer, Dieter Oesterhelt

**Affiliations:** Department of Membrane Biochemistry, Max-Planck-Institute of Biochemisty, Am Klopferspitz 18, Martinsried 82152, Germany; E-Mail: oesterhe@biochem.mpg.de

**Keywords:** genome annotation, Gold Standard Protein, Halobacteria, halophilic archaea, manual curation

## Abstract

Genome annotation errors are a persistent problem that impede research in the biosciences. A manual curation effort is described that attempts to produce high-quality genome annotations for a set of haloarchaeal genomes (*Halobacterium salinarum* and *Hbt. hubeiense*, *Haloferax volcanii* and *Hfx. mediterranei*, *Natronomonas pharaonis* and *Nmn. moolapensis*, *Haloquadratum walsbyi* strains HBSQ001 and C23, *Natrialba magadii*, *Haloarcula marismortui* and *Har. hispanica*, and *Halohasta litchfieldiae*). Genomes are checked for missing genes, start codon misassignments, and disrupted genes. Assignments of a specific function are preferably based on experimentally characterized homologs (Gold Standard Proteins). To avoid overannotation, which is a major source of database errors, we restrict annotation to only general function assignments when support for a specific substrate assignment is insufficient. This strategy results in annotations that are resistant to the plethora of errors that compromise public databases. Annotation consistency is rigorously validated for ortholog pairs from the genomes surveyed. The annotation is regularly crosschecked against the UniProt database to further improve annotations and increase the level of standardization. Enhanced genome annotations are submitted to public databases (EMBL/GenBank, UniProt), to the benefit of the scientific community. The enhanced annotations are also publically available via HaloLex.

## 1. Introduction

Protein function assignments in public databases suffer from severe errors. It has been estimated that incorrect assignments of a specific function may affect as many as 30% of the proteins, and may even exceed 80% for certain protein families [1,2]. Genomes are commonly subjected to automatic annotation procedures by computational annotation robots. As these procedures build on the information provided in public databases, errors in the database may be “propagated, leading to a potential transitive catastrophe” [3]. Overall, it was estimated that the relative error rate is increasing over time [2]. Error propagation could be substantially reduced if annotations are copied only from those proteins which themselves have been functionally characterized. Such proteins are referred to as “Gold Standard Proteins” [4,5]. The SwissProt section of UniProt is a rich source for Gold Standard Proteins, generated by extensive expert curation [6,7], but suffers from a considerable incompleteness. Protein sets in the UniProt HAMAP system are based on Gold Standard Protein seeds [8]. InterPro and its partner databases also provide information about functionally characterized proteins in their annotation [9]. An important and reliable resource for metabolic enzymes is the KEGG database [10]. The arCOG database provides annotation information for archaeal ortholog sets [11,12]. Genome annotations may suffer from additional problems such as (a) missing gene annotations; (b) incorrect start codon assignments; or (c) invalid handling of disrupted genes (also referred to as pseudogenes).

We have sequenced and annotated five haloarchaeal genomes, *Halobacterium salinarum* strain R1 [13], *Natronomonas pharaonis* [14], *Natronomonas moolapensis* [15], as well as the *Haloquadratum walsbyi* strains HBSQ001 and C23 [16,17]. Genome annotation included a detailed reconstruction of metabolic pathways [18] as a prerequisite for whole-genome metabolic modeling [19,20,21]. We have also participated in genome annotation of *Haloferax volcanii* [22], *Natrialba magadii* [23], and *Halobacterium hubeiense* [24].

Here we describe the strategy of our genome annotation efforts. Using Gold Standard Proteins as the preferred basis for function annotation makes our approach resistant against the transitive catastrophe of database errors. Enhanced annotation is also achieved by systematic consistency checking between more than 10 haloarchaeal genomes. Annotations are regularly reconciled with those in public databases. By providing regular updates and feedback to major public databases, our effort is of benefit for a larger research community.

## 2. Experimental Section

Data management using HaloLex. All data are managed in the HaloLex genome annotation system in annotation mode [25]. Beyond providing basic functionalities, such as a genome viewer and information container, key features are (a) a “region” status referring to protein existence, start codon assignment, and gene disruption; (b) a ‘function’ status referring to function assignments; (c) internal comments that allow to describe considerations underlying decision making during the manual curation effort; (d) tools which are specifically tailored to revise start codon assignments; and (e) tools for management of disrupted genes.

Most genomes contain disrupted genes, which are a challenge to gene calling. Many disrupted genes cannot be represented as a single contiguous open reading frame. We have updated the HaloLex “protein” formalism to allow for multiple fragments (equivalent to a discontiguous reading frame). As an example, a protein that has been targeted by a transposon is represented by a set of two ORFs, one for the region preceding the transposon, the other for the region following the transposon. The protein sequence is obtained by independent translation of the fragments, which are then concatenated into a single protein sequence. The resulting sequence reflects the ancestral gene and frequently shows a full-length alignment to homologs. Disrupted genes have a “pseudogene” flag and the protein name contains the term “(nonfunctional)”.

Standard bioinformatic tools and public databases. A significant part of our effort is built on the BLAST suite of programs [26]. For annotation issues we extensively access the UniProt database, especially the SwissProt (curated) subsection [6,7], the HAMAP system of UniProt [8], and InterPro annotations [9]. Metabolic data are accessed via the KEGG database [10]. Transposons are analyzed with the help of the ISFinder database [27]. Homology searches are performed against UniProt, the NCBI “nr” database and the ‘Halobacteria’ subset of the NCBI whole-genome shotgun contigs [28].

Specific function annotation is based on Gold Standard Proteins. As a basic strategy, we allow only experimentally characterized homologs (Gold Standard Proteins) as a valid source of specific function assignments. The identification of such homologs is based on database analyses (mainly using the SwissProt section of UniProt and InterPro annotations), and on extensive literature searches. We commonly access PubMed (http:www.ncbi.nlm.nih.gov/pubmed), including its search and “cited for” functionalities.

Missing genes, start codon assignment checking, and spurious ORF calls. To search for missing genes, proteins from selected haloarchaeal genomes are compared to the genome under study using blastP and tblastN [26]. Proteins with a higher tblastN than blastP score are candidates for identification of missing genes. Such hits are manually validated [25] and newly identified genes are post-predicted using the sixframe translator functionality of HaloLex.

Start codon assignments are consistent with our extensive characterization of the *N*-termini using proteomic data for *Hbt. salinarum* and *Nmn. pharaonis* [29,30]. We extensively apply homology-based start codon assignment checking as previously described [25].

Spurious gene calls are open reading frames that are unlikely to code for a protein. Such spurious ORFs are especially frequent in high-GC genomes like those of halophilic archaea [13,25,30]. In HaloLex, such calls are invisibly retained as “spurious ORF” (region status “del”) but can be accessed when appropriate, e.g., when searching for missing genes or displaying non-coding genome regions.

Annotation consistency checking. Consistency checking is applied to a set of haloarchaeal genomes. The list of all bidirectional best blast pairs is computed, the vast majority of which represent orthologs. The annotation (protein name, gene name, EC number) is compared to validate consistency. Cases where annotation differences cannot be avoided are handled by explicitly recording the manually assigned annotation in an “exceptions file”. The few bidirectional best blast pairs, considered to represent paralogs or even casual blast matches, rather than orthologs, are also recorded in the “exceptions file” and are excluded from consistency checking.

Public database correlation. For genomes subjected to public database correlation, a broker file is generated, holding annotation information (protein name, gene name, EC number) and sequence information (protein sequence length, and genome coordinates). Each HaloLex ORF is correlated to the associated UniProt entry (by database section and code) and to the EMBL feature (by EMBL accession and locus tag). For each combination of ORF and database, an annotation status and a sequence status is assigned, based on the “current” annotation. The status is “ok” when the annotation is consistent, either because all data are identical or because data represent merely style differences and can be automatically interconverted. The latter is necessary as, e.g., protein names are capitalized in UniProt while they are lowercase in HaloLex and in EMBL.

When a new version of a database becomes available, it is compared to the broker file with revised annotations being added as “modified” data. All “modified” data are evaluated, triggering appropriate downstream processing (by updating the status), and turned into “current” data. Several processing scenarios are described in the Supplementary Text. Illustrated is (a) a HaloLex revision which is transferred to EMBL and further to UniProt and (b) a revision in a single UniProt entry which triggers updating of a complete ortholog set in HaloLex with subsequent transfer to EMBL and UniProt.

## 3. Results and Discussion

### 3.1. The General Strategy Applied to Protein Function Annotation and Examples of Protein Misannotation

We aim to provide high quality annotations for a set of haloarchaeal genomes with respect to the sequences themselves (see below) and to the assignment of the biological function. The genomes currently under survey are listed in Table 1.

Protein function is represented by protein name, gene assignment, and EC number. We attempt to follow accepted standards as much as possible. We aim to provide a correct function annotation, free of “false negatives” (incomplete annotations) but also free of “false positives” (overannotations, *i.e.*, invalid assigning a specific protein function when at maximum a general assignment is supported by the available evidences). It has been reported that for several protein families more than 80% of database entries may carry an invalid specific function assignment [2], identifying overannotation as one of the major source of database errors.

The overannotation problem can be illustrated by Hmuk_0137 from *Halomicrobium mukohataei*, a protein which is erroneously annotated as “cobyrinic acid ac-diamide synthase”. Enzymes with this function catalyze a step in *de novo* cobalamin biosynthesis, a metabolic pathway that is lacking in *Hmc. mukohataei*. This organism does not have orthologs to any of the other at least 10 enzymes that catalyze the conversion of precorrin-2 to cobyrinate a,c diamide. Even worse, the erroneous annotation as “cobyrinic acid ac-diamide synthase” is not only assigned to Hmuk_0137 but to a set of six paralogs. All of these proteins have an assigned InterPro domain IPR002586 that had been named “Cobyrinic a,c-diamide synthase” up to 2012. The underlying pattern is, however, very general and also identifies other proteins which are only very distantly related, including proteins from the ParA/MinD family. Most halophilic archaea code for several paralogs from this protein family, including the six overannotated proteins from *Hmc. mukohataei*. While the assignment of IPR002586 to these proteins is correct and points to distant sequence homology, and while naming of the domain as “cobyrinic acid ac-diamide synthase” is valid because this enzyme is one of the representatives having this domain, it was invalid that annotation robots picked an InterPro domain header as protein name, ignoring that this domain is assigned to many sets of non-orthologous proteins. When we pointed out to InterPro that this domain name is a source of a severe overannotation, InterPro renamed the domain to “CobQ/CobB/MinD/ParA nucleotide binding domain”. However, renaming of an InterPro domain does not trigger correction of protein names that are based on outdated versions of domain headers. Thus, there are still many overannotated proteins in UniProt/TrEMBL resulting even in erroneous annotation of proteins from newly annotated genomes, again illustrating the “transitive catastrophe”.

**Table 1:** Genomes under annotation survey.

Organism	Contribution ^1^	Proteins ^2^	Locus Tags	UniProt Organism Code	EMBL Accessions	Reference
*Halobacterium salinarum* strain R1	seq, anno	2845	OE_	_HALS3	AM774415-AM774419	[13]
*Natronomonas pharaonis*	seq, anno	2864	NP_	_NATPD	CR936257-CR936259	[14]
*Natronomonas moolapensis*	seq, anno	2881	Nmlp_	_NATM8	HF582854	[15]
*Haloquadratum walsbyi* strain HBSQ001	seq, anno	2874	HQ_	_HALWD	AM180088-AM180089	[16]
*Haloquadratum walsbyi* strain C23	seq, anno	2995	Hqrw_	_HALWC	FR746099-FR746102	[17]
*Haloferax volcanii*	anno	4040	HVO_	_HALVD	CP001953-CP001957	[22]
*Natrialba magadii*	anno	4295	Nmag_	_NATMM	CP001932-CP001935	[23]
*Halobacterium hubeiense*	anno	3437	Hhub_	-	-	[24]
*Haloferax mediterranei*	3rd party	3859	HFX_	_HALMT	CP001868-CP001871	[31]
*Haloarcula marismortui*	3rd party	4290	rrnAC, rrnB, pNG	_HALMA	AY596290-AY596298	[32]
*Haloarcula hispanica*	3rd party	3859	HAH_	_HALHT	CP006884-CP006886	[33]
*Halohasta litchfieldiae*	3rd party	3350	halTADL_	-	-	[34]

^1^ Contribution refers to participation of our group in genome sequencing (seq), annotation (anno), or no participation (3rd party); ^2^ Based on HaloLex.

**Figure 1:**
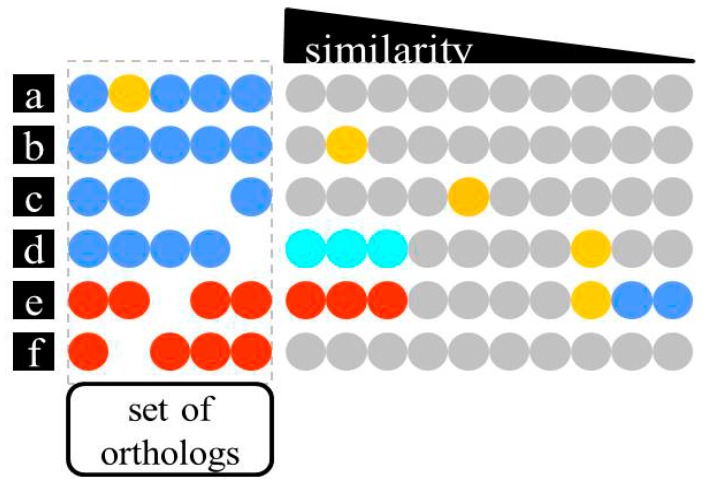
Schematic illustration of homology-based function assignment, based on Gold Standard Proteins. Each row represents a set of orthologs from the haloarchaeal genomes under survey (colored dots). These may be absent from some of the genomes (empty places). Other proteins are represented with decreasing sequence similarity. Gold standard proteins (yellow) are proteins which have been reported to be functionally characterized. Supposed orthologs of Gold Standard Proteins are indicated in blue, proteins that are not considered to be orthologs in red. Grey dots are homologs for which no decision has been attempted. (**a**) a protein from the set of haloarchaeal genomes has been experimentally characterized; (**b**) a closely related Gold Standard Protein; and (**c**) a more distantly related Gold Standard Protein have been characterized and are considered orthologous to the haloarchaeal proteins; (**d**) the haloarchaeal proteins are rated to be orthologs in a transitive way. While they are too distant to the Gold Standard Protein to support orthology directly, there are “bridging” proteins (light blue) that are close enough to both; (**e**) a Gold Standard Protein is too distant to be considered an ortholog and a “bridging” homolog cannot be identified. Only a general annotation can be used in this case; (**f**) none of the homologs could be identified as a Gold Standard Protein.

Being aware of overannotation as a major source of database errors, we decided that specific functional annotations need to be strictly connected to experimental data (as illustrated in Figure 1). Only proteins which themselves have been experimentally characterized (Gold Standard Proteins) are accepted as valid information donors for homology-based annotation transfer. Applying this principle, manual curation includes the task of identifying at least one functionally characterized homolog for every set of orthologs encoded by one of the haloarchaeal genomes under review. However, it has to be decided if this homolog is closely enough related to be considered an ortholog and is the most closely related homolog in the genome under study. Decision-making is an important aspect of manual curation, which seems resistant to automation. As different protein families evolve at a different rate and differ in their tendency to adopt new functions, there is no general cutoff which can be applied [35]. Even in a manual curation effort, this step is a potential source of annotation errors.

Additional biological knowledge also needs to be considered, as illustrated by the following two examples: (1) The oxidative decarboxylation of pyruvate to acetyl-CoA is ferredoxin-dependent in *Hbt. salinarum (halobium)* [36,37] and other halophilic archaea. In contrast, the oxidation of pyruvate to acetyl-CoA is catalyzed by an NAD-dependent protein complex in bacteria. (A parallel situation exists for conversion of alpha-ketoglutarate to succinyl-CoA.) *Hbt. salinarum* codes for homologs to the subunits of the bacterial NAD-dependent pyruvate dehydrogenase complex, which are unrelated to the experimentally characterized, ferredoxin-dependent enzymes. Whatever the function of the homologs to the bacterial NAD-dependent complex is, they cannot be involved in the oxidation of pyruvate to acetyl-CoA (or alpha-ketoglutarate to succinyl-CoA) as this would imply NAD-dependent conversions which have been excluded by experimental analysis [36]. Based on this evidence, only a general annotation is assigned in HaloLex, while the subunits of the *Hbt. salinarum* complex are wrongly annotated as pyruvate dehydrogenase, not only via automatic genome annotation pipelines, but even in the KEGG database. The specific function of one of the homologous complexes in *Hfx. volcanii* has been identified, showing that this complex is involved in isoleucine degradation [38] but the specific substrates of the other complexes remain currently enigmatic. (2) The enzyme 5,10-methylenetetrahydromethanopterin reductase (EC 1.5.98.2) from *Methanothermobacter thermautotrophicus* (MTH_1752) has been experimentally characterized [39] and has close homologs in halophilic archaea (e.g., HVO_1937 from *Hfx. volcanii*). In UniProt, HVO_1937 is currently annotated as a methanopterin-specific enzyme according to the *Methanothermobacter* homolog. However, this is invalid as halophilic archaea do not contain the coenzyme methanopterin as the coenzyme of C1 carbon metabolism but instead use tetrahydrofolate [40]. Thus, we annotate this protein as “probable 5,10-methylenetetrahydrofolate reductase”, consistent with knowledge about haloarchaeal biology. UniProt has been informed about this annotation problem via their feedback system and thus probably will have corrected the annotation in one of the next releases.

In rare cases, we assign specific functions to proteins even without a closely related experimentally characterized homolog. Examples are predictions based on distant homologs which are supported by e.g., gene neighborhood analysis, substrate assignments based on detailed 3D structure model building, or close but uncharacterized homologs that have their specific function assigned using the UniProt HAMAP system.

If an experimentally characterized homolog cannot be identified or is too distant to be considered an ortholog, we assign at maximum a general function to the protein. For general function assignments we prefer names like “GNAT family acetyltransferase” or “DUF2267 family protein”. In some cases, names start with the terms “homolog to” (e.g., homolog to 4-hydroxy-tetrahydrodipicolinate synthase). We use this when we are convinced that the corresponding protein does **not** have the named function (otherwise we would name it by its function without the term “homolog to”).

### 3.2. Identification of Experimentally Characterized Homologs (Gold Standard Proteins)

“Gold Standard Proteins” in the SwissProt section of UniProt contain publications that describe experimental characterization, tagged by terms like “Function” or “Characterization” in the “Cited for:” field. The “evidence” links in the FUNCTION section refer to these publications and corresponding entries can be selected by using “scope:function” as search term.

**Figure 2:**
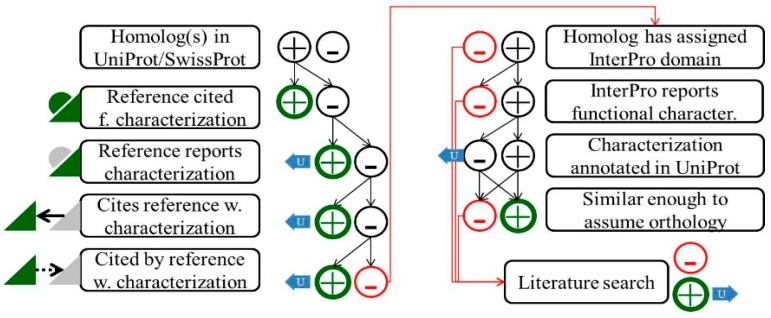
Schematic illustration of the procedure to identify publications describing experimental protein characterization. Two major routes to identify Gold Standard Proteins are illustrated. (**Left column**): Homologs in UniProt/SwissProt are identified by a blastP search. Many of the UniProt/SwissProt entries represent manually curated Gold Standard Proteins, with a reference describing (green triangle) and “cited for” (green half-circle) experimental characterization. If this approach is successful, the evaluated protein can be annotated accordingly (green circle “plus”). UniProt/SwissProt entries lacking such a reference may have been annotated via the HAMAP system, which may allow identification of a Gold Standard Protein ortholog using the “scope:function” search option (not illustrated). On the other hand, the UniProt/SwissProt entry may be incomplete insofar as a reference reports functional characterization but is not flagged accordingly (grey half circle). The haloarchaeal protein is annotated according to this Gold Standard Protein and an update of the UniProt entry is proposed via the UniProt feedback system (blue arrow). The publication may report just the sequence (grey triangle) and cite another publication for experimental characterization. Frequently, sequencing precedes function assignment. The publication reporting experimental characterization commonly cites the sequencing paper and might be detected e.g., via the “cited by” functionality in PubMed. (**Right column**): If the UniProt/SwissProt attempt is not successful, homologs in UniProt/TrEMBL are checked for an assigned domain in InterPro. The InterPro annotation may report experimental protein characterizations. If the underlying UniProt entry already reports this characterization (and thus is in UniProt/SwissProt), this protein must be too distant to be considered an ortholog. Otherwise it would already have been identified via the UniProt/SwissProt approach (left column). If characterization of the protein is not reported in UniProt (which will trigger feedback), the protein sequence is used to identify potential haloarchaeal orthologs. If the protein under evaluation is considered an ortholog, it is annotated accordingly. If the InterPro approach also fails, a more extended literature search may be launched. If a Gold Standard Protein ortholog can be identified by literature search, the corresponding UniProt entry is identified and evaluated for orthology. Haloarchaeal orthologs are annotated accordingly (and UniProt/InterPro, which must be incomplete if this approach is required, are informed via feedback). If a literature search is also not successful, only a general annotation is possible.

To decide if a haloarchaeal ortholog set can have a specific function assigned, we need to identify a Gold Standard Protein homolog and an associated publication describing functional characterization in order to be consistent with our general annotation strategy. As illustrated in Figure 2, we first compare the protein sequence against UniProt/SwissProt using blastP, attempting to retrieve a functionally characterized protein with a link to experimental characterization. If the UniProt/SwissProt database would be complete and perfect, implementation of our general annotation strategy would have been a trivial task. As can be seen in the scheme, more time-intensive approaches are necessary when a Gold Standard Protein homolog is not easily retrievable via UniProt/SwissProt (Figure 2). We systematically report such cases back to UniProt (which has already resulted in many database improvements).

Domain annotations accessible via InterPro were found to be another promising option to identify publications that report functional protein characterization (Figure 2). During our efforts, we also provided substantial feedback to the InterPro team when we identified a publication reporting experimental protein characterization but which was not yet included in the domain annotation. We expect that, via interdatabase communication, this information will also help to improve other databases like KEGG or the emerging Combrex database.

### 3.3. Annotation Consistency Checking

Orthologs have an identical function and thus should also have an identical function annotation (protein name, gene name, EC number). For a narrow taxonomic branch like the halophilic archaea, bidirectional best blast can be used as a simple but efficient representation of orthologs. We thus decided to compare the functional annotation of all bidirectional best blast pairs for the set of haloarchaeal genomes under study (Table 1). An in-house script (discrepancy checker) lists all discrepancies, triggering manual curation of the corresponding ortholog pairs.

In many cases, non-identitity of the annotation cannot be avoided. An example is *cheY*/*cheY1* pair from *Hbt. salinarum* (OE_2417R, *cheY*) and *Nmn. pharaonis* (NP_2102A, *cheY1*, 72% sequence identity). While there is only a single *cheY* gene in *Hbt. salinarum*, there is a paralog in *Nmn. pharaonis* (NP_0516A, *cheY2*, 50% sequence identity), making the usage of gene serial numbers necessary. In this case, the assigned gene symbols are listed in an “exceptions file” to indicate that they are considered valid. When the annotation of an ortholog pair differs but both annotations are listed in the “exceptions file”, the discrepancy checker does not report this case. In an attempt to generate fully consistent annotations, all reported discrepancies between 12 haloarchaeal genomes have been resolved by manual curation, leading to an empty discrepancy checker report.

While most of the bidirectional best blast pairs represent orthologs, this is not always the case. An example pair are the retinal proteins *Hqr. walsbyi* HQ_1017A and *Nmn. pharaonis* NP_4834A. HQ_1017A has been described as deeply branching bacteriorhodopsin II [16], but is also called “middle rhodopsin” [41] due to its special characteristics. NP_4834A is sensory rhodopsin which mediates the phototactic response [42]. *Hqr. walsbyi* is non-motile and thus has lost all motility-associated genes, including sensory rhodopsin. *Nmn. pharaonis* suffered a species-specific loss of the bacteriorhodopsin gene, which may be related to its alkaliphilic lifestyle. Due to the lack of the closely related orthologs, a more distant retinal protein is retrieved as best blast hit, which in this case happens to result in a non-orthologous bidirectional best blast pair. Bidirectional best blast pairs that are rated to be paralogs or casual blast matches are recorded in the “exceptions file” and are excluded from annotation consistency checking.

### 3.4. Comparison to Public Databases

**Figure 3:**
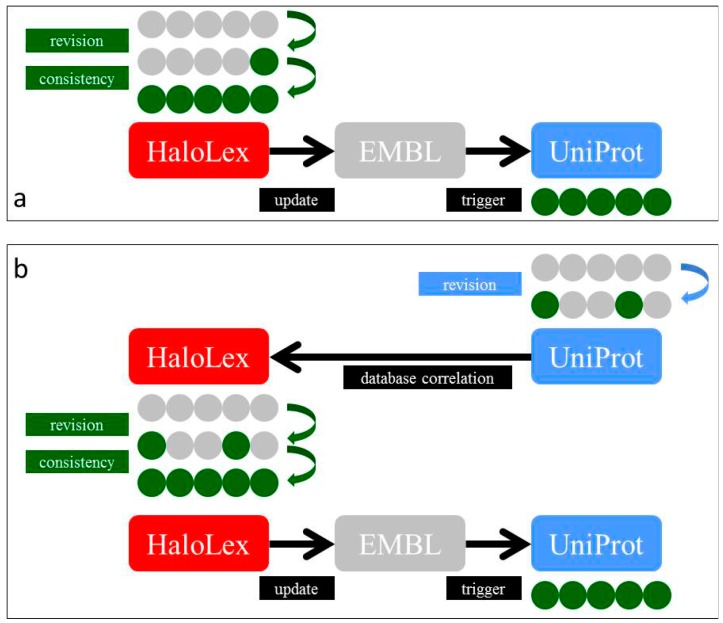
Schematic illustration of the interaction with public databases. (**a**) Updating of one member of an ortholog set in HaloLex triggers updating of all the other members, which is validated via consistency checking. Updated genome features are submitted to EMBL. These updates are forwarded to UniProt via inter-database communication, leading to updated UniProt entries a few releases later. (**b**) Updated and improved annotation at UniProt may affect only a subset of the sequences from the ortholog set. The UniProt update is detected by the database correlation approach and triggers updating of the corresponding HaloLex entries and all haloarchaeal orthologs from the genomes under survey. The improved annotation is forwarded to EMBL (as in (a)) and may lead to updating of additional proteins in UniProt.

The HaloLex annotation is correlated with UniProt and with EMBL (and thus also GenBank). Correlation with UniProt is a two-way process: (a) annotation improvements in HaloLex will enhance the associated UniProt entries (Figure 3a); (b) UniProt staff regularly improves annotations. When a protein from our set of haloarchael genomes is modified, this will trigger manual curation in order to also enhance the HaloLex annotation (Figure 3b). Correlation with EMBL is only a one-way process as EMBL/GenBank do not themselves enhance annotations. We enhance the annotation of genomes under our responsibility by submitting updated features when a sufficient number of modifications has accumulated. Due to inter-database communication, the EMBL update will immediately trigger a GenBank update and will also, after some time, trigger a UniProt update.

To achieve correlation with public databases, we use broker files that contain the current information and are enriched with modified data when revisions are encountered. All modified data are then subjected to manual evaluation, which may trigger updates in HaloLex or EMBL/UniProt revisions as further detailed in Methods and the Supplementary text. This approach would not be possible without the efforts taken by UniProt staff to name proteins in a highly standardized way. Additional public databases could be incorporated into the system. We recently initiated a correlation to the arCOG and KEGG databases.

Annotation updates are submitted for the haloarchaeal genomes sequenced by our group and for some of those cooperative genome projects where we have been assigned to maintain the genome annotation. Annotation updates for genomes where we have not been involved in sequencing and/or annotation are not possible due to EMBL/GenBank policies that allow only authors to make such modifications. As a consequence, errors persist in the database unless the authors themselves correct them. Even UniProt is not able to remove those annotation errors from the TrEMBL database, which they are aware of, unless the original authors revise their genome annotation (which—according to our experience—occurs only extremely rarely). There clearly is a need to reconsider database policies so that the error rate can be reduced.

### 3.5. Sequence Annotation Checking

Sequence annotation checking (managed via the “region” status in HaloLex) has several aspects: (a) start codon assignment checking; (b) identification of “spurious ORFs”, *i.e.*, open reading frames which do not code for a protein (such spurious ORFs are especially frequent in high-GC genomes such as those of halophilic archaea) [25,30]; (c) analysis of disrupted genes; and, (d) genes that may be missing in the ORF set and need to be post-predicted.

Incorrect start codon assignments may severely interfere with subsequent experimental analysis of the corresponding proteins. It is obvious that assignment of an internal Met as the translation start may cause problems. Cloning of the corresponding gene region into an expression vector will lead to an incomplete protein, which may be instable or nonfunctional. On the other hand, the assignment of an incorrect start codon upstream of the *in vivo* start may interfere with functional characterization. This is exemplified by *Hfx. volcanii* HVO_2177. A ubiquitin-like covalent protein modification (“sampylation”) has been detected in this organism [43]. Initially, only two proteins were found to be covalently attached, the homologous proteins SAMP1 (HVO_2619) and SAMP2 (HVO_0202). HVO_2177, which is homologous to SAMP1 and SAMP2, was found **not** to be covalently attached to proteins [43]. Later, however, it was found that the start codon has been misassigned, leading to a protein which is too long. The shortened protein expressed from the corrected start codon was found to be covalently attached to proteins and HVO_2177 is now annotated as SAMP3 [44].

We have analyzed our extensive set of proteomic data for identification of *N*-terminal peptides [29,30]. In addition, we extensively used homology-based start codon checking to validate/correct start codon assignments [25]. We found that Glimmer, one of the commonly used gene predictors, has a 30% error rate with respect to start codon assignments when applied to genomes with high GC content [29]. In addition, even though the genomes of *Hbt. salinarum* strains R1 and NRC-1 [45] show only marginal chromosomal sequence differences, start codon assignment discrepancies were found for 20% of the genes [13]. We developed a script that classifies blast alignments of bidirectional best blast pairs with respect to the “configuration” at the *N*-terminus. Some of these configurations are associated with a high probability that at least one of the start codons is misassigned (e.g., when the Met at position 1 of a protein sequence aligns with an internal Met of the other sequence). Such cases are subjected to manual curation.

While some genomes (e.g., *Hbt. salinarum* strain NRC-1, *Har. marismortui*) have remained static even though extensive sets of likely start codon misassignments have been reported [13,25], other groups have retrieved the improved annotation from HaloLex and have subsequently revised the genome annotation (e.g., *Hfx. mediterranei*). The proteins from the latter organism have been systematically compared to their orthologs from *Hfx. volcanii*, leading to start codon reassignments in both organisms.

### 3.6. Disrupted Genes

By definition, complete prokaryotic genomes cannot contain protein fragments. However, genes may be inactivated leading to gene remnants which resemble protein fragments. There is a relatively small list of well-defined biological processes which lead to gene inactivation: (a) targeting by transposons and other mobile genetic elements; (b) point mutations that convert a sense codon into an in-frame stop codon; (c) mutations leading to insertion/deletion of one or few bases, frequently causing frameshifts; (d) larger deletions or genome rearrangements which may truncate genes at either or both ends, may remove long internal regions of the gene, or may lead to chimaeric genes. Many of these disrupted genes cannot be represented as single ORFs but need to be represented by joining multiple coding regions. The HaloLex data model allows representation of all types of disrupted genes. Unfortunately, the corresponding information cannot be represented in the public databases which only allow for a rudimentary description of inactivated prokaryotic genes.

A special problem is the presence of genes that have been targeted by transposons. In such cases, the *N*-terminal part of the ORF fragment is translated (in silico and eventually also *in vivo*) up to the genome/transposon junction. At this point, translation continues into the transposon up to the first in-frame stop codon. Dependent on the insertion details (orientation, frame), this may lead to a chimeric protein with a relatively long C-terminal region derived from one of the six frames of the transposon (very rarely including the transposase gene itself). If the same transposon integrates into another gene with identical insertion details, this will generate a second chimeric protein with an identical C-terminal region. Annotation robots may misunderstand this as sequence homology and may invalidly transfer the annotation from one to the other gene.

Handling of the C-terminal regions is more variable: (a) if the C-terminal region is long enough and the protein has an internal Met residue (or a Val residue encoded by GTG), this can be misannotated as a start codon (as exemplified below); (b) the transposon may have an ATG or GTG trinucleotide which happens to be in-frame with the C-terminal region of the ORF. This may lead to a chimaeric sequence starting within the transposon and continuing into the C-term ORF region beyond the transposon/genome junction; (c) If the C-terminal region is short or devoid of in-frame ATG or GTG codons, the ORF may not be annotated. As long intergenic regions are atypical in prokaryotic genomes, gene finders may translate a different frame in this region, leading to ORPHans which may even show signs of “sequence conservation” between genomes (especially if the disrupted gene is highly conserved).

One such example is a gene that is interrupted by an isopositioned transposon (ISH1) in both strains of *Hbt. salinarum* (the transposon sequences themselves differ slightly). In strain R1, the ancestral gene (OE_1059R) has been reconstructed, the ISH element being inserted close to codon 102. The reconstructed protein sequence shows 68% identity to HALDL1_00590 from *Halobacterium sp.* DL1 and also 68% identity to HFX_4100 from *Hfx. mediterranei*. In strain NRC-1, only the C-terminal region is annotated (VNG0034H), starting with internal Met-145.

The representation of such inactivated genes in public databases depends on the way they are annotated. Labelling of such a gene as “inactivated” seems biologically correct. This is translated to the CDS qualifier /pseudo in EMBL and securely ensures that the protein translation is **not** present in UniProt (e.g., searching for OE_1059R results in no hit). When, however, an invalid partial translation product is produced but not tagged as disrupted (as is the case for VNG0034H), then this is considered by EMBL as a “regular” gene (CDS). Such a gene fragment is included as a regular protein in UniProt (VNG0034H is Q9HSX6). Upon superficial analysis, this may be taken as evidence for an “improved” (because less incomplete) genome annotation in strain NRC-1 compared to strain R1. In addition, according to EMBL requirements, the “CDS” coordinates of OE_1059R must be given as 29913-31570, thus covering and including the integrated transposon ISH1 (with its transposase gene). Only a “tolerated” misc_feature annotation allows representation of this disrupted gene in a biologically meaningful way, representing the reconstructed ancestral gene.

## 4. Conclusions

We describe an effort for a high-quality annotation of a set of haloarchaeal genomes. Among those are two reference organisms, *Hbt. salinarum* (represented by strain R1) and *Hfx. volcanii*. The annotation of *Hbt. salinarum* strain NRC-1 [45], the classical genome of halophilic archaea, is covered by our approach as nearly all its genes are represented in strain R1 with an identical protein sequence (once start codon misassignments are corrected). *Hfx. volcanii*, one of the reference organisms for archaea [46] is intensely studied by many laboratories from the haloarchaeal community, which is in part due to the extremely well developed genetic system available for this organism.

Key to our annotation concept is the restriction to Gold Standard Proteins as the only valid data source for homology-based annotation. Not only have we retrieved this information from public databases, especially UniProt/SwissProt, but we also have contributed to the improvement of that database by supplying a substantial amount of feedback. Improved database retrieval mechanisms that directly highlight Gold Standard Proteins would largely facilitate to adopt our annotation strategy. We hope that our improved annotation will also be transferred to other haloarchaeal genomes, including the large number of haloarchaeal genomes that have been recently reported [47], or to other high-level datasets like arCOG [11,12].

Advanced bioinformatic methods have a tremendous potential to advance biological knowledge (see e.g., [35]). Preferably, bioinformatic predictions should be backed up by experimental analyses, as has been done for archaeosortase [48,49]. Efficient large-scale screening techniques, such as the transposon insertion mutant library recently developed for *Hfx. volcanii* are also promising to advance our knowledge [50]. Nevertheless, sound biochemical analysis remains important in the process of fundamental discovery. Using this approach, the long sought-after oxidative pentose phosphate pathway has finally been identified in archaea by analyses with the model organism *Haloferax volcanii* [51].

It is evident that a single small annotation team cannot ensure a perfect annotation. We welcome feedback to this publication, such as reporting persistent omissions and errors in annotations, which will allow us to further improve the annotation of this set of haloarchaeal genomes. If it were possible to transfer our improved annotation to other haloarchaeal genomes, this may boost the quality of haloarchaeal genome annotation in general, to the benefit of the scientific community.

## Supplementary Files

Supplementary File 1
