# Supplementary material for: A Manual Curation Strategy to Improve Genome Annotation: Application to a Set of Haloarchael Genomes
Source: Life (Basel). 2015 Jun 2;5(2):1427–44. doi: 10.3390/life5021427 (PMC4500146; doi:10.3390/life5021427)
Supplement: Supplementary File 1 [file life-05-01427-s001.pdf]

OPEN ACCESS

life

ISSN 2075-1729

www.mdpi.com/journal/life

Supplementary Information

A Manual Curation Strategy to Improve Genome Annotation: Application to a Set of Haloarchael Genomes. *Life* 2015, *5*, 1427-1444

Friedhelm Pfeiffer * and Dieter Oesterhelt

Department of Membrane Biochemistry, Max-Planck-Institute of Biochemisty, Am Klopferspitz 18, Martinsried 82152, Germany; E-Mail: oesterhe@biochem.mpg.de

* Author to whom correspondence should be addressed; E-Mail: fpf@biochem.mpg.de; Tel.: +49-89-8578-2323.

A typical database update cycle consists of several steps. Described are (A) an update cycle initialized by HaloLex (Supplementary Table S1) and (B) an update cycle initialized by UniProt (Supplementary Table S2).

Table S1. A HaloLex-triggered database update.

Action	Broker File Data	EMBL Status	UniProt Status
before update	"current data"	ok	ok
load updated HaloLex data	add "modified" data		
evaluate modified data	"modified" => "current"	todo_submit	Wait4Transfer(pending)
submit revised features to EMBL		UpdReq(*date*)	Wait4Transfer(*date*)
download revised EMBL entry			
load updated EMBL data	add "modified" data		
evaluate modified data	"modified" => "current"	isRevised	
run status checker script		ok	
download new UniProt release			
load updated UniProt data	add "modified" data		
evaluate modified data	"modified" => "current"		isRevised
run status checker script			ok
after update	updated "current" data	ok	ok

(A1) The annotation in HaloLex is enhanced, e.g., because a publication describing experimental protein characterization is identified and is considered applicable to the haloarchaeal homologs, rated to be orthologs. (A2) At the next HaloLex loading, the script adds the improved annotation to the broker file, leading to an EMBL update request ("todo_submit") for the next update cycle. The anticipated UniProt update via inter-database communication is recorded ("Wait4Transfer"). (A3) When a sufficient number of changes have been made, a revised EMBL feature table is prepared for the genome and submitted to EMBL. The EMBL status is changed ("UpdReq") and the submission date is recorded for both, EMBL and UniProt. (A4) Once the revised EMBL genome becomes publically available, the new version is loaded into the Broker file and for all EMBL-modified proteins the status is updated ("isRevised"). If modifications are encountered which have not been triggered (as indicated by lack of the status "UpdReq"), the entry will be flagged for subsequent evaluation. (A5) The resulting broker file is analyzed by an in-house script (StatusChecker), updating status "isRevised" to "ok" if the annotation has become consistent. At this stage, neither the status "UpdReq" nor the status "isRevised" should persist. A persistant status "UpdReq" indicates a failure to make the update and a persistent status "isRevised" indicates a data discrepancy. Both cases trigger further analysis. (A6) UniProt releases appear about once per month and are loaded into the broker file when available. Once EMBL has implemented the genome update, corresponding modifications become visible in UniProt some releases later and are detected as modified data. Modifications which have been triggered (status Wait4Transfer) receive status "isRevised", which is then updated to "ok" if the resulting annotation is consistent. Again, persistence of the status "Wait4Transfer" indicates a failure to update and persistence of "isRevised" indicates a data discrepancy. Update "failures" may indicate that the entry in UniProt is above the level that allows automatic updating. In this case, update requests are submitted directly to UniProt via their feedback system. At the end, the annotation has been updated in all databases and should be consistent between databases.

(B1) Modifications that have been triggered by UniProt are detected as modified data, not being associated with status Wait4Transfer. These are flagged for subsequent manual evaluation ("UniProtBetter"), eventually leading to an improved HaloLex annotation. (B2) This improvement will be harmonized among the set of genomes under survey and triggers further EMBL updates. (B3) Improved UniProt entries have a higher annotation status. For proteins from the corresponding organisms (species_A), only EMBL will be updated. Subsequent data transfer to UniProt is not possible but also not necessray as the annotation is already up to date. For organisms which were not subjected to revision by UniProt (species_B), the consistency efforts within HaloLex will lead to an annotation update in UniProt, resolving some of the database inconsistencies.

Table S2. A UniProt-triggered database update.

Action	Species A		Species B	
	**Broker File Data**	**UniProt Status**	**Broker File Data**	**UniProt Status**
before update	“current” data	ok	“current” data	ok
download new UniProt release				
load updated UniProt data	add “modified” data		(unchanged)	
evaluate modified data	“modified” => “current”	UniProtBetter		ok
update in HaloLex				
load updated HaloLex data	add “modified” data		add “modified” data	
evaluate modified data	“modified” => “current”	isRevised	“modified” => “current”	Wait4Transfer(pending)
run status checker script		ok		Wait4Transfer(pending)
(EMBL update as in SupplementaryTable1)				Wait4Transfer(*date*)
download new UniProt release				
load updated UniProt data	(unchanged)		add “modified” data	
evaluate modified data		ok	“modified” => “current”	isRevised
run status checker script		ok		ok
after update	updated “current” data	ok	updated “current” data	ok

© 2015 by the authors; licensee MDPI, Basel, Switzerland. This article is an open access article distributed under the terms and conditions of the Creative Commons Attribution license (http://creativecommons.org/licenses/by/4.0/).
